# Fulminant Type 1 Diabetes Mellitus Leading to Diabetic Ketoacidosis and Mesenteric Ischemia With Necrosis Following Pembrolizumab Administration: A Case Report

**DOI:** 10.7759/cureus.76687

**Published:** 2024-12-31

**Authors:** Mayu Ueki, Takeshi Fukuda, Kenta Oue, Takuma Wada, Toshiyuki Sumi

**Affiliations:** 1 Department of Obstetrics and Gynecology, Osaka Metropolitan University Graduate School of Medicine, Osaka, JPN; 2 Department of Obstetrics and Gynecology, Osaka City University Graduate School of Medicine, Osaka, JPN

**Keywords:** acute mesenteric ischemia (ami), fulminant type 1 diabetes mellitus, immune-checkpoint inhibitors, immune-related adverse events (iraes), pembrolizumab side effect, programmed death-1 (pd-1) inhibitors, severe diabetic ketoacidosis, uterine cervical cancer

## Abstract

Immune checkpoint inhibitors (ICIs), such as pembrolizumab, have revolutionized cancer therapy but can lead to severe immune-related adverse events (irAEs). We present a case of fulminant type 1 diabetes mellitus (T1DM) with diabetic ketoacidosis (DKA) and mesenteric ischemia in a 78-year-old woman with recurrent stage IIIC1 cervical cancer treated with pembrolizumab. Thirty-four days after initiating a pembrolizumab-containing regimen, she presented with vomiting, severe hyperglycemia, metabolic acidosis, and strongly positive urine ketones. Laboratory findings confirmed complete insulin deficiency, leading to a diagnosis of fulminant T1DM and DKA, requiring intensive insulin therapy. Despite treatment, her condition rapidly deteriorated, with worsening DKA, hyperglycemia, gastrointestinal bleeding, and extensive mucosal necrosis identified through endoscopy and imaging. The patient ultimately progressed to septic shock and died the same day. Fulminant T1DM is characterized by abrupt β-cell destruction and rapid DKA onset. In this case, DKA likely contributed to mesenteric ischemia, a severe vascular complication. This is the first reported case of fulminant T1DM, DKA, and mesenteric ischemia following pembrolizumab. The case underscores the importance of vigilance, early recognition, and multidisciplinary management of irAEs to prevent fatal outcomes.

## Introduction

Cervical cancer remains a significant global health concern for women. In 2022, it was estimated that there were 661,021 new cases and 348,189 deaths attributed to the disease worldwide. This places cervical cancer as the fourth most commonly diagnosed cancer and the fourth leading cause of cancer-related mortality among women [1]. Recent advancements in treatment strategies have expanded beyond conventional cytotoxic chemotherapy to include immune checkpoint inhibitors such as pembrolizumab, nivolumab, atezolizumab, durvalumab, cemiplimab, avelumab, and ipilimumab [2].

Among these, pembrolizumab, a programmed death-1 (PD-1) immune checkpoint inhibitor, has emerged as a key therapeutic option for cervical cancer. TheKEYNOTE-826trial provided compelling evidence supporting the efficacy of pembrolizumab in this context. This pivotal study evaluated the combination of pembrolizumab with chemotherapy as a first-line treatment for patients with persistent, recurrent, or metastatic cervical cancer. The findings demonstrated significant improvements in both overall survival and progression-free survival compared to the placebo plus chemotherapy group [3].

Despite their clinical benefits, immune checkpoint inhibitors (ICIs) are associated with a distinct spectrum of immune-related adverse events (irAEs) that differ considerably from those seen with traditional cytotoxic chemotherapy. These irAEs can affect multiple organ systems, including the liver, gastrointestinal tract, endocrine glands, skin, and lungs [4]. This distinct adverse event profile underscores the importance of specialized monitoring and management strategies tailored to ICIs.

Herein, we report a case of fulminant type 1 diabetes mellitus (T1DM) leading to diabetic ketoacidosis (DKA) and mesenteric ischemia with necrosis following pembrolizumab administration.

## Case presentation

**Figure 1:**
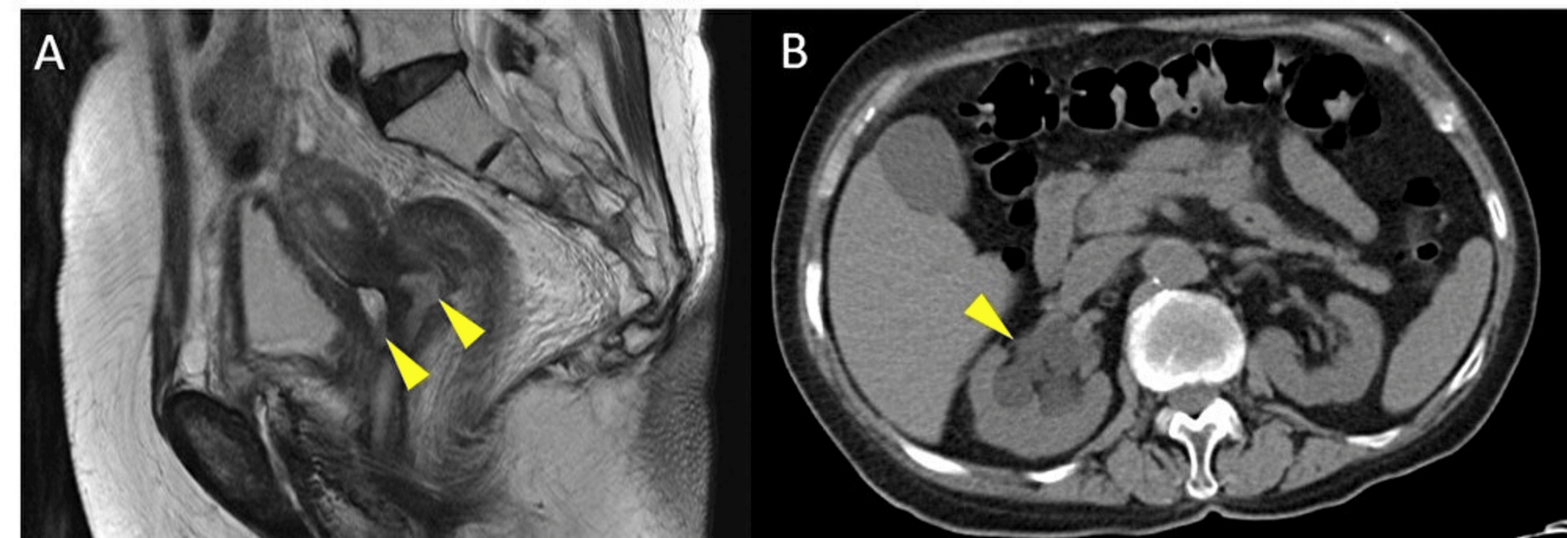
Magnetic resonance imaging and computed tomography A: MRI shows recurrent tumors in the uterine cervix and the vesicouterine pouch (arrowheads); B: CT shows hydronephrosis in the right kidney (arrowhead)

A 78-year-old woman was diagnosed with stage IIIC1 (T1b2N1M0) cervical cancer of squamous cell carcinoma and underwent concurrent chemoradiotherapy. Her past medical history included type 2 diabetes mellitus, for which she was taking sitagliptin at 25 mg per day. Her glycosylated hemoglobin (A1c) level was approximately 7% (normal range: 4.6-6.2%). The concurrent chemoradiotherapy consisted of external beam radiation therapy (36.0 Gy) and intracavitary brachytherapy (24.0 Gy) using a remote after-loading system, combined with weekly administration of cisplatin (CDDP) at 40 mg/m² for a total of five cycles. After the treatment, the tumor completely disappeared, and the evaluation was determined to be a complete response. Three years post-treatment, pelvic magnetic resonance imaging revealed recurrent tumors in the uterine cervix and vesicouterine pouch, and abdominal computed tomography (CT) revealed hydronephrosis in the right kidney caused by the recurrent tumors (Figure 1).

**Figure 2:**
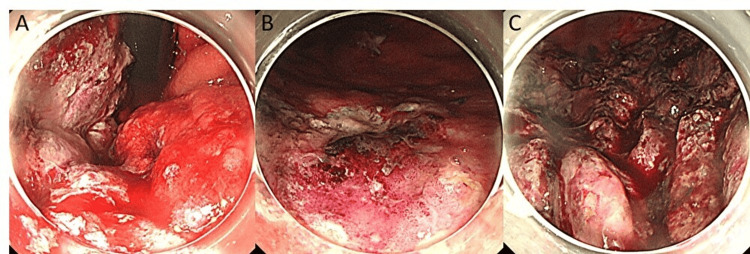
Images of upper gastrointestinal endoscopy Upper gastrointestinal endoscopy reveals extensive mucosal necrosis caused by ischemic changes due to blood flow disturbances. A: gastric fundus; B, C: greater curvature of the gastric body

Her serum creatinine level was elevated to 1.30 mg/dL (normal range: 0.46-0.79 mg/mL). A nephrostomy was performed on the right kidney, improving her serum creatinine level to 0.77 mg/dL. Subsequently, chemotherapy consisting of paclitaxel (175 mg/m²), carboplatin (AUC 5), and pembrolizumab (200 mg/body) was initiated for the recurrence. The glycosylated hemoglobin (HbA1c) level prior to starting treatment was 6.7%. On day 34, after chemotherapy administration, the patient experienced vomiting, and her blood glucose level was 539 mg/dL (normal range: 73-109 mEq/l). Although chemotherapy is typically administered at 21-day intervals, the initiation of the next cycle was delayed due to the patient's general fatigue. The arterial blood pH was 7.159 (normal range: 7.35-7.45 ng/mL), the anion gap was 14 mEq/l (normal range: 10-14 mEq/l), urine ketones were strongly positive (3+), and C-peptide immunoreactivity was 0.25 ng/mL (normal range: 0.80-2.50 ng/mL). She exhibited Kussmaul respiration. She was diagnosed with DKA due to fulminant T1DM and started on intravenous fluids along with a continuous infusion of Humulin® R at four units per hour. Later, it was confirmed that anti-glutamic acid decarboxylase (anti-GAD)antibodies were negative. On the following day, her condition deteriorated further. She exhibited drowsiness with a Glasgow Coma Scale (GCS) score of 13 (E3, V4, M6), and her blood glucose level rose to 873 mg/dL. The arterial blood pH dropped to 7.13 (normal range: 7.35-7.45), indicating worsening diabetic ketoacidosis, and mechanical ventilation was initiated. In addition, she presented with dark red vomitus and melena, which were indicative of upper gastrointestinal bleeding. Her hemoglobin level decreased from 9.9 g/dL to 7.7 g/dL. Upper gastrointestinal endoscopy revealed extensive mucosal necrosis due to ischemic changes associated with blood flow disturbance, primarily involving the gastric fundus and the greater curvature of the gastric body (Figure 2).

**Figure 3:**
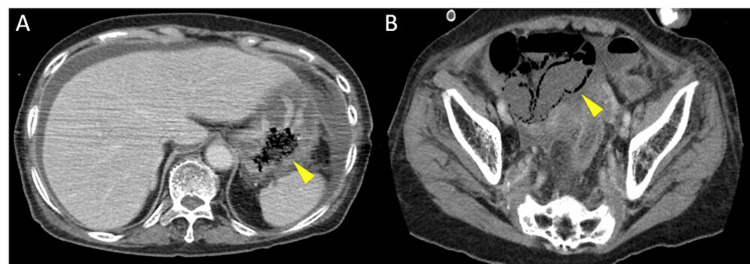
Contrast-enhanced abdominal computed tomography Contrast-enhanced CT images show areas of contrast defects suggestive of ischemia or necrosis (arrowheads). A: gastric fundus and the upper body of the stomach; B: small intestine

Oozing bleeding was observed throughout the necrotic area. Contrast-enhanced abdominal CT demonstrated areas of contrast defects suggestive of ischemia and necrosis involving the gastric fundus, the upper body of the stomach, and segments of the small intestine (Figure 3). No thrombus was identified on the contrast-enhanced CT.

The definitive treatment for intestinal necrosis is surgical resection of the necrotic segments. However, the patient's general condition was deemed too compromised to tolerate surgical intervention. Following thorough discussions with the patient's family, the best supportive care was implemented. Unfortunately, the patient succumbed to septic shock later the same day.

## Discussion

Immune checkpoint inhibitors have become integral to the treatment of various cancers, including cervical cancer. Pembrolizumab, a humanized immunoglobulin G4 (IgG4) monoclonal antibody targeting the PD-1 receptor, is an effective ICI for cervical cancer. By blocking PD-1, pembrolizumab enhances T-cell activity, thereby promoting a robust immune response against cancer cells. Common adverse events include fatigue, rash, pruritus, and diarrhea, while less frequent irAEs such as hypothyroidism and colitis have also been reported [5]. Despite their efficacy in a subset of cervical cancer patients, ICIs are associated with a wide spectrum of irAEs, which differ mechanistically and clinically from those caused by traditional cytotoxic chemotherapy. Among these irAEs, the development of T1DM is a rare but serious complication. The incidence of ICI-induced T1DM is estimated at approximately 3.5% [6], with DKA reported in over 60% of cases - a potentially life-threatening condition that typically arises within weeks of initiating ICI therapy [7]. DKA associated with ICI-induced T1DM progresses rapidly, often resulting in the depletion of endogenous insulin secretion within three weeks of onset [6]. The pathophysiology involves T-cell-mediated destruction of pancreatic β-cells due to disrupted PD-1 and programmed death-ligand 1 (PD-L1) signaling. At present, insulin therapy remains the primary treatment for ICI-induced T1DM [8].

DKA is also linked to an increased risk of mesenteric ischemia and necrosis, a rare but potentially fatal complication. This condition can occur in both mild and severe cases of DKA and may present as either occlusive or non-occlusive mesenteric ischemia, with dehydration being a significant risk factor [9,10]. Clinical manifestations often include persistent abdominal pain, vomiting, and signs of sepsis. Management strategies include medical stabilization, revascularization, and surgical resection of necrotic tissue [9].

**Figure 4:**
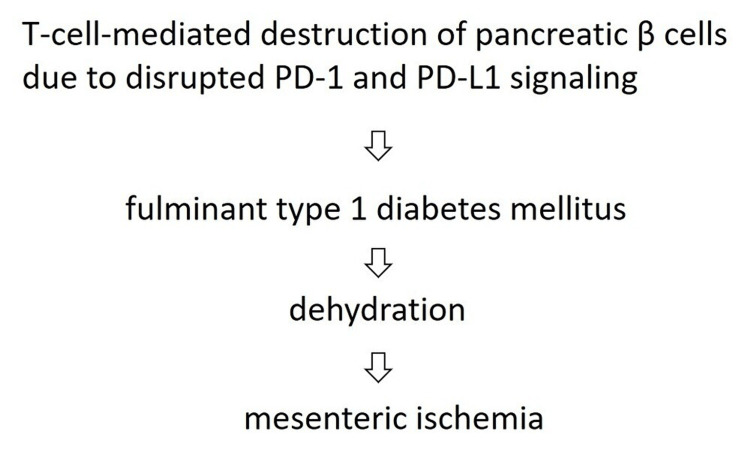
Possible underlying mechanism of mesenteric ischemia PD-1: programmed death-1; PD-L1 - programmed death-ligand 1

In the present case, T1DM developed after a single dose of pembrolizumab, leading to DKA, which was complicated by mesenteric ischemia and necrosis. The possible underlying mechanism is illustrated in Figure 4.

While surgical resection of necrotic tissue is an effective treatment option, the patient's poor general condition rendered surgery unfeasible. This ultimately resulted in sepsis and death. This case underscores the importance of prompt recognition and management of severe irAEs, particularly those involving metabolic and vascular complications.

## Conclusions

We report a case of fulminant T1DM leading to diabetic ketoacidosis DKA and mesenteric ischemia with necrosis following a single dose of pembrolizumab. To the best of our knowledge, this is the first documented case of such severe complications occurring after just one dose of pembrolizumab. This case highlights the critical need for vigilance regarding the broad spectrum of irAEs associated with ICIs. Early recognition and timely intervention are essential, particularly for rare but life-threatening conditions such as intestinal necrosis secondary to DKA. This report serves as a cautionary reminder of the potential for fulminant T1DM, DKA, and mesenteric ischemia following pembrolizumab administration, even after a single dose. Furthermore, this case highlights the importance of multidisciplinary approaches to effectively manage ICI-induced adverse events and optimize patient outcomes.
